# Influence of Child Factors on Health-Care Professionals’ Recognition of Common Childhood Mental-Health Problems

**DOI:** 10.1007/s10826-016-0475-9

**Published:** 2016-07-01

**Authors:** Delia A. Burke, Hans M. Koot, Amber de Wilde, Sander Begeer

**Affiliations:** Department of Clinical Developmental Psychology and EMGO Institute of Health and Care Research, VU University Amsterdam, Van der Boechorststraat 1, 1081 BT Amsterdam, The Netherlands

**Keywords:** Mental-health problems, Children, Demographic characteristics, Health-care professionals, Symptoms, Recognition

## Abstract

Early recognition of childhood mental-health problems can help minimise long-term negative outcomes. Recognition of mental-health problems, needed for referral and diagnostic evaluation, is largely dependent on health-care professionals’ (HCPs) judgement of symptoms presented by the child. This study aimed to establish whether HCPs recognition of mental-health problems varies as a function of three child-related factors (type of problem, number of symptoms, and demographic characteristics). In an online survey, HCPs (*n* = 431) evaluated a series of vignettes describing children with symptoms of mental-health problems. Vignettes varied by problem type (Attention-Deficit/Hyperactivity Disorder (ADHD), Generalised Anxiety Disorder (GAD), Autism Spectrum Disorder (ASD), Conduct Disorder (CD) and Major Depressive Disorder), number of symptoms presented (few and many), and child demographic characteristics (ethnicity, gender, age and socio-economic status (SES)). Results show that recognition of mental-health problems varies by problem type, with ADHD best recognised and GAD worst. Furthermore, recognition varies by the number of symptoms presented. Unexpectedly, a child’s gender, ethnicity and family SES did not influence likelihood of problem recognition. These results are the first to reveal differences in HCPs’ recognition of various common childhood mental-health problems. HCPs in practice should be advised about poor recognition of GAD, and superior recognition of ADHD, if recognition of all childhood mental-health problems is to be equal.

## Introduction

Early recognition of mental-health problems in children can help minimise long-term negative outcomes for the child and his or her environment (Kessler et al. 2003; Nelson et al. 2003). Recognition of mental-health problems precedes and differs from diagnosis; it involves health-care professionals’ (HCPs) *initial* evaluation of and concern about symptoms presented by a child (McConaughy 2013). If symptoms are not recognised as indicators of potential mental-health problems during first contact with a HCP, the diagnostic process (including referral, evaluation and diagnosis) is unlikely to be initiated (Hawkins-Walsh 2001). However, the factors which influence recognition of child mental-health problems are largely unexamined. Though there is some indication that a child’s ethnic characteristics bias HCPs’ recognition of mental-health problems (Burke et al. 2015; Froehlich et al. 2007), fundamental questions remain, particularly whether HCPs recognise different mental-health problems to an equal extent, whether differential recognition may be related to the number of symptoms presented and to other demographic features.

All types of HCPs working in child and adolescent health-care see children at varying stages on their pathway to treatment, and are required to make judgements that affect long-term patient outcomes (Stiffman et al. 2004). Whilst HCPs’ goals are to evaluate, diagnose and treat a patient’s problem (McConaughy 2013), the process of evaluating a patient, beginning with recognition, is far from straight-forward. Several factors may unintentionally influence HCPs’ ability to recognise mental-health problems, and eventually lead to missed- (Cassidy and Jellinek 1998; Farmer and Griffiths 1992), under- (Burke et al. 2015), over- (Bruchmüller et al. 2012), or mis-diagnosis (Dossetor 2007). For example, not all symptoms are unique to specific disorders; mood problems may occur in the context of anxiety as well as depression. In addition, demographic characteristics, such as age, may be key to informing HCPs about appropriateness of a behaviour (American Psychological Association (APA) 2013). In order to ensure accurate and timely recognition of childhood mental-health problems, the factors that influence HCPs’ recognition must be understood. The type of problem, number of presenting symptoms, and basic demographic characteristics, including ethnicity, gender, age and socio-economic status (SES), are candidate child factors to be examined, given their known influence on diagnosis (APA 2013) later in the diagnostic process.

The type of problem may be particularly influential in HCPs’ recognition. However, we know remarkably little about the basic question whether some childhood mental problems are easier to recognise than others, even among the most commonly occurring problems. There are, nevertheless, reasons to expect differences in the recognisability of different childhood mental-health problems. Some mental-health problems are more prevalent than others, which is likely to increase the HCPs’ exposure to these problems and, in turn, their ability to recognise them on first sight (Matson and Kozlowski 2011). For example, based on the current estimated prevalence rates for Attention-Deficit/Hyperactivity Disorder (ADHD; 3.5 %), Generalised Anxiety Disorder (GAD; 1.4 %), Conduct Disorder (CD; 3 %), Major Depressive Disorder (MDD; 1.7 %) (Merikangas et al. 2009) and Autism Spectrum Disorders (ASD; 1 %) (Centers for Disease Control (CDC), n.d.), recognition of ADHD could be expected to be best. Similarly, the visibility of or disruption caused by problems may affect their recognisability. For example, externalising disorders, like ADHD or CD, may be better recognised because they are more disruptive to the environment than others and therefore attract more attention than internalising disorders like anxiety and depression (Mesman and Koot 2000). However, no study to date has sought to systematically compare potential variation in HCPs’ recognition of different common child and adolescent mental-health problems.

The number of presenting symptoms may be another factor relevant for recognition. Greater numbers of presenting symptoms are likely to increase problem salience and to help in the process of eliminating other possible problems (McConaughy 2013). Particularly since some problems, such as anxiety and depressive disorders, share symptoms (Allgulander 2006; Brown 1997; Brown et al. 2001). However, the number of symptoms required for recognition may vary for different types of problem. For example, ADHD is characterised by two distinct domains, specifically, inattention and hyperactivity-impulsivity (APA 2013), and whilst there is a long list of criteria to be met before a child can receive a formal *diagnosis* of ADHD, it is plausible that a mere two symptoms representing those core domains are sufficient for HCPs to *recognise* ADHD. The same argument could apply to the core characteristic domains of ASD (qualitative limitations in communication and social interaction, and repetitive behaviours; APA 2013). On the other hand, although MDD is characterised by a depressed mood (APA 2013), the presence of a depressed mood as such may be less likely to trigger recognition of a clinical problem since depressed mood is a normal reaction to certain circumstances (Horwitz and Wakefield 2007). HCPs may therefore need to see multiple symptoms of MDD before they recognise it as a possible mental-health problem. Identifying which mental-health problems are recognisable at the presentation of just a few symptoms and which problems require many symptoms before they are recognisable, will help highlight mental-health problems that are at risk of being overlooked in clinical practice.

In clinical practice it is standard procedure for patients to provide demographic characteristics, including ethnicity, gender, age and SES. Demographic characteristics can be consciously or unconsciously processed (Devine 1989; Kinzler et al. 2010) and can also influence attitudes without conscious awareness (Nosek et al. 2002). During evaluation and diagnosis, demographic information is duly considered by HCPs (APA 2013); furthermore, the effect of demographic characteristics such as ethnicity on access, referral, evaluation, and diagnosis has been extensively examined (e.g., Malgady 1996; Mandell et al. 2002; Yeh et al. 2002). However, how demographic characteristics influence HCPs’ *recognition* of different childhood mental-health problems has received insufficient attention. This is an important issue to study since whilst demographic information should be weighted during evaluation and diagnosis, it should not influence recognition to the extent that problems are ultimately overlooked. For instance, children from ethnic-minority groups are less likely to have symptoms of autism recognised than their majority-group peers despite both groups presenting identical symptoms (Begeer et al. 2009; Burke et al. 2015). ADHD and autism are less likely to be recognised in girls than boys, even when both present equal symptoms (Froehlich et al. 2007; Russell 2011), and ADHD and autism seem to be more likely to be recognised in children from low and high SES backgrounds respectively (Cuccaro and Wright 1996). Surprisingly, research investigating the effect of a child’s age on HCPs’ recognition of any disorder could not be found. Research is needed to address this omission and to further examine the effects of ethnicity, gender and SES on HCPs’ ability to recognise different mental-health problems. The present study examined the influence of the type of problem, the number of symptoms presented, the demographic characteristics of a child, as well as their interactive effects, on HCPs’ recognition during evaluation of information concerning children and adolescents with symptoms of mental-health problems. The first aim of the study was to compare HCPs’ recognition of five common child/adolescent disorders (Attention-Deficit/Hyperactivity Disorder (ADHD), Generalised Anxiety Disorder (GAD), Autistic Disorder (ASD), Conduct Disorder (CD) and Major Depressive Disorder (MDD)). The second aim was to examine HCPs’ recognition of those problems when they present few and many symptoms. The final aim of the study was to explore the differential effect of basic demographic characteristics (ethnicity, gender, age and SES) on HCPs’ recognition of the mental-health problems listed above. The following hypotheses were tested: (1) Recognition of ADHD and CD may be greater than that of each of the other disorders since the prevalence of ADHD and CD in children is higher than the other childhood mental-health problems evaluated here (Merikangas et al. 2009). In addition, ADHD and CD are externalising disorders with more disruptive characteristics than internalising disorders and therefore recognised easier (Mesman and Koot 2000), and ADHD may have been primed for recognition due to media attention in recent years (Matson and Kozlowski 2011). Whilst ASD is lowest in prevalence (CDC, n.d.), it is a disorder with some externalising features, and has also recently received increased media attention. For these reasons, recognition of ASD was expected to be poorer than that of ADHD and CD, but better than the internalising disorders MDD and GAD. Analyses regarding the order in which GAD and MDD are recognised were exploratory. (2) Based purely on the number of presenting symptoms, vignettes with many symptoms were expected to be better recognised than those with few symptoms. (3) In terms of the effects of demographic characteristics, ethnic-minority group children were expected to be less likely to have ASD recognised than their majority group counterparts (Begeer et al. 2009; Burke et al. 2015), whilst ADHD and ASD were expected to be recognised more often in boys than in girls (Froehlich et al. 2007; Russell 2011), and in children from a high SES in comparison to low SES background (Cuccaro and Wright 1996). Further differential analyses of effects of demographic characteristics were exploratory.

## Method

### Participants

Participants were 431 child and adolescent HCPs (89 % women) employed in the Netherlands who were either recruited through their place of employment or through an affiliation with health-care societies and associations. Participants responded to an advertisement published on their work or society website, or in an employee newsletter. Of the participants, 125 (29 %) were psychologists, 91 (21 %) were paediatricians, 91 (21 %) were pedagogues (professionals with a master’s degree in child-development and education, who work in mental health and child-care settings), 17 (4 %) were psychiatrists, 52 (12 %) were social-workers and 34 (8 %) were teachers and school mentors. The remainder of participants (21; 5 %) were non-practicing (health) care professionals, employed for example, as professors or policy makers. See Table 1 for participants’ descriptive statistics. Ethical approval for the study was granted by the ethical committee of the VU University, Amsterdam.

**Table 1:** Participants’ demographic characteristics and descriptive statistics

Characteristic	*N*	**%**
Gender		
Male	47	10.90
Female	384	89.10
Age		
18–24	10	2.32
25–39	159	36.89
40–59	222	51.51
60+	39	9.05
Unknown/missing	1	.23
HCP type		
Psychologist	125	29.00
Paediatrician	91	21.11
Pedagogue	91	21.11
Psychiatrist	17	3.94
Social-worker	52	12.07
Teacher/school-mentor	34	7.90
Non-practicing health-care	21	4.87
HCP experience		
0–5 years	106	24.59
5–10 years	92	21.35
10–15 years	74	17.17
15–20 years	53	12.30
20 + years	106	24.59
Ethnicity		
Dutch majority	376	87.24
Non-Dutch minority	51	11.83
Unknown/missing	4	.93

### Procedure

An advertisement, entitled *Evaluating School Children with Mental*-*Health Problems,* was published on employee websites and in online newsletters, and outlined the study’s interest in the role of HCPs during initial evaluation of children. The advertisement included a link to the online survey. Before the survey began, participants were presented with a screen with brief instructions which explicitly stated that vignettes did not provide all information required to make a *diagnosis,* but they would evoke a first impression and that is what we were interested in. Consent to use collected data was obtained at this point. The first page of the survey collected demographic information about the participant. In the pages that followed, participants were shown 10 vignettes, one per page. Each vignette was presented in combination with an open question regarding *recognition*. Once participants proceeded to the next page, they were not able to scroll back and alter previous responses. To finish, information about participants’ job and experience as a HCP was requested.

### Measures

HCPs evaluated a series of vignettes describing children with symptoms of different mental-health problems. Only by using a standardised experimental analogue design can vignettes of various mental-health problems with equivalent symptomatology be created, thereby allowing comparison of HCPs’ recognition of these problems. Unlike real-life diagnostic evaluations, whereby contextual factors may be impossible to control, a simulated HCP-child evaluation creates optimal experimental conditions. Specifically, all independent variables are systematically varied and randomly presented to ensure they are evaluated equally, instructions and questions are standardised, descriptions of each disorder with few and many symptoms vary *only* by demographic characteristics and finally, descriptions of each demographic category are held constant. The latter means, for example, that where children are described as having a low SES background, low SES does not vary in magnitude. Similarly, where children are described as belonging to an ethnic-minority group, the immigrant generation described is stable across group categories.

The following variables were manipulated systematically in each vignette viewed by participants: problem type, number of symptoms, and the demographic characteristics. The full combination of problem type (5) x number of symptoms (2) x ethnicity (5) x gender (2) x age (2) x SES (2) resulted in a total of 400 possible vignettes. From this total number of vignettes, 40 surveys were created, each containing ten vignettes. Every survey was identical with regards to its composition of variable combinations. Parents and teachers were described as informants of children in all of the vignettes (see Appendix for vignette examples). Of the ten vignettes included in each survey, there were two vignettes describing each problem and two of each ethnicity. The remaining dichotomous categories (few vs. many symptoms; male vs. female; child vs adolescent; and low vs high SES) were distributed evenly, with five vignettes representing each category. All vignettes were counterbalanced both across and within each of the 40 surveys to avoid ordering effects, and surveys were randomly presented to participants. All vignettes used in the surveys are available from the first author.

#### Type of Problem

Each vignette described a child presenting symptoms of one of the following five mental disorders: ADHD, GAD, ASD, CD and MDD. The DSM-IV-TR (APA 2000) criteria for each of these disorders were used to compose the vignettes with appropriate symptoms. At the time of the study, DSM-5 (APA 2013) was yet to be published; DSM-IV is therefore most likely to have informed HCPs’ education on mental health problems. Vignettes of each disorder were developed in three stages: In the first stage, symptoms to be included in the descriptions were selected from the DSM criteria. During the second stage, psychologists and pedagogues (*n* = 5) who regularly work with children were consulted for advice regarding age-appropriate expression of the selected criteria. In the final stage, a pilot study (*n* = 24) was conducted amongst HCPs and confirmed that, irrespective of other variables, the types of problem described in the vignettes were recognisable above chance level. No further changes were made to the vignettes after the pilot study.

#### Number of Symptoms

The number of symptoms presented in the vignettes varied to include ‘few’ or ‘many’. Few-symptom vignettes described 5 symptoms whilst many-symptom vignettes described 10 symptoms. Symptoms included in vignettes describing each disorder were taken from each disorder’s corresponding DSM-IV-TR criteria. Although criteria for each of the disorders differ, it was possible to systematically select symptoms based on similarities in the structure of each disorder’s criteria. Specifically, each disorder includes a number of *necessary* criteria that are required to be met, and a single criterion, with a number of *possible* symptoms. For each of the disorders described in the vignettes, symptoms were chosen to meet the necessary criteria first. The remaining symptoms were then randomly selected from the possible symptoms. To illustrate, when creating a GAD vignette with 5 symptoms, the first three symptoms were taken from GAD’s three necessary criteria and the remaining 2 symptoms were randomly selected from the disorders’ possible symptoms. For a vignette describing GAD with 10 symptoms, the first three symptoms were taken from the necessary criteria but then *7* remaining symptoms would be selected from the possible symptoms.

It is important to note that *possible symptoms* are clustered into domains for some disorders but not for others. For example, possible symptoms for ADHD are clustered into the two domains (*inattention* and *hyperactivity*-*impulsivity)*. In ASD, possible symptoms are clustered into three domains (*social interactions*, *communications* and *restrictive behaviours*). In GAD, CD and MDD, on the other hand, possible symptoms are listed under one domain, whereby no single symptom is more typical than the other. For disorders where possible symptoms are clustered into domains, symptoms were randomly selected from each of the domains. For example, when creating an ADHD 5 symptom vignette, the first two symptoms were taken from ADHD’s two necessary criteria; the remaining 3 symptoms were alternately selected at random from the two domains, *inattention* and *hyperactivity*-*impulsivity.* For a vignette with 10 symptoms, the procedure was identical but then 8 remaining symptoms would be alternately selected from the two domains. If the number of remaining symptoms could not be taken evenly from each of the domains, certain domains were more heavily represented in vignettes than others. In that case, symptoms from the most persistent domains of the respective disorder were chosen to be most represented, in order to reflect a prototypical case. In ADHD, symptoms from the inattention domain are most persistent throughout its development and course, with hyperactivity diminishing throughout development. In ASD, symptoms from impaired *social interactions* and *communications* domains are most persistent throughout its development and course (APA 2013).

#### Ethnicity

The vignettes differed by ethnic background of the child to include Dutch majority cases, western (European) minority cases (English) and non-western (Moroccan, Turkish and Indian) minority cases. Moroccan and Turkish minority children reflect the largest non-western minority groups in the Netherlands. English and Indian children were included to avoid transparency of this manipulation. The ethnic background varied independently of the vignette content and was reflected in the child’s name and description of their country of origin.

#### Gender

Vignettes differed by children’s gender. Gender was never explicitly mentioned but reflected in the appropriate pronoun.

#### Age

Age of the child presented varied and was categorised as child or adolescent. Children were described as of primary-school age or younger (3–10 years). Descriptions of adolescents ranged between ages 11 and 17 years.

#### SES

SES was varied in the description of the child’s parents’ job and was categorised as low or high. The Netherlands Central Bureau of Statistics (Centraal Bureau voor de Statistiek (CBS) 2010) provides an extensive list of current jobs which are categorised ordinally, with categories ranging from 1 (elementary jobs) to 8 (scientific jobs). Jobs from categories 1 and 8 were used in vignettes to describe children with a low and high SES background respectively.

#### Recognition

HCPs evaluated children described in the vignettes using a single item. An open question asked participants “Please briefly indicate whether you consider the described vignette as a cause for concern. If yes, what do you think is the matter with the child?” Participants responded in their own words. Responses to this item were coded dichotomously as having recognised the disorder described in the vignette (1), or having made no reference/erroneously referencing an unrelated disorder/problem (0). Responses were coded ‘recognised’ when HCPs named the disorder described in the vignette, or referred to a disorder that is categorised (in the DSM-IV-TR) under the same *subheading* as the described disorder. The latter were included as ‘recognised’ because disorders categorised under one subheading share common feature(s) (APA 2000), that disorders under other subheadings do not. To illustrate, responses to vignettes describing ASD were coded as ‘recognised’ if a participant explicitly named ASD or referred to a disorder categorised under the subheading *Pervasive developmental disorders*, such as Asperger’s Disorder or PDD-NOS. Similarly, responses to vignettes describing MDD were coded as ‘recognised’ if a participant explicitly named MDD or referred to a disorder categorised under the subheading *Depressive disorders*, such as Dysthymic Disorder or Depressive Disorder-NOS. The DSM-IV-TR does not divide anxiety disorders under subheadings, indicating at least a single common feature across all anxiety disorders (Shear et al. 2007). For this reason, responses to vignettes describing GAD were coded as ‘recognised’ if a participant explicitly named GAD or referred to any other anxiety disorder.

### Data Analyses

Reliability analyses, using the kappa statistic (*κ*), were calculated in SPSS statistics 21 (IBM corp 2012) to determine agreement of coding amongst two raters. Twenty percent of vignettes were randomly selected from each described disorder for coding by a second, independent rater. The inter-rater reliability was found to be *κ* = 0.91 (*p* < .001) for coding responses to ADHD vignettes, *κ* = .98 (*p* < .001) for GAD vignettes, *κ* = 1.00 (*p* < .001) for ASD vignettes, *κ* = 0.93 (*p* < .001) for CD vignettes, and *κ* = 1.00 (*p* < .001) for MDD vignettes, thereby indicating near perfect agreement.

MLwin version 2.30 (Rabash et al. 2014) was utilised for all analyses to control for nested data within participants. Recognition was analysed using multi-level logistic regression with random intercept. The only level two variable to be included in analyses was *HCP type.* This was added to all analyses as a covariate, in order to control for the heterogeneity of HCPs, since it was not within the scope of this study to examine their potential differences. The type of mental-health problem, number of symptoms, ethnicity, gender, age and SES were level one variables. All variables were first included in one main effects model to test their individual association with recognition. In a second step, two and three-way interaction terms were added to the model. Contrasts were made between each of the disorders with few and many symptoms but only significant results are reported. Finally, all *p* values were adjusted for multiple comparisons using the Bonferroni method.

## Results

### Effect of Problem Type and Number of Symptoms on Recognition

The type of problem presented in the vignettes influenced the frequency of recognition by the HCPs, *χ*^*2*^ (4) = 57.67, *p* < .001. Vignettes describing children with ADHD elicited greater recognition than those describing GAD, *χ*^*2*^ (1) = 43.79, *p* < .001, ASD, *χ*^*2*^ (1) = 40.56, *p* < .001, CD, *χ*^*2*^ (1) = 29.85, *p* < .001, and MDD, *χ*^*2*^ (1) = 33.99, *p* < .001. There were no differences in recognition between GAD, ASD, CD and MDD. See Table 2 for descriptive statistics and Table 3 for inferential statistics.

**Table 2:** Recognition (%) of mental-health problems by type (ADHD, GAD, ASD, CD, MDD), number of symptoms (few, many) and age group (children, adolescents)

	ADHD	GAD	ASD	CD	MDD
Symptoms					
Few	65	63	29	57	48
Many	78	53	88	64	72
Age					
Child	68	62	67	60	46
Adolescent	75	55	51	62	75
Children					
Few	63	64	44	59	30
Many	73	60	89	60	62
Adolescent					
Few	67	62	15	55	68
Many	83	47	87	69	81
Overall					
	72	58	59	60	60

*ADHD* Attention deficit hyperactivity disorder, *GAD* Generalised anxiety disorder, *ASD* Autistic disorder, *CD* Conduct disorder, *MDD* Major depressive disorder

**Table 3:** Multi-level logistic regression coefficients and odds ratios for HCP recognition

		95 % CI for odds ratio		
	B (SE)	Lower	Odds ratio	Upper
*Problem type*				
ADHD vs. GAD	.75 (.11)***	1.89	2.12	2.33
ADHD vs. ASD	.72 (.11)***	1.84	2.06	2.28
ADHD vs. CD	.63 (.11)***	1.64	1.86	2.08
ADHD vs. MDD	.66 (.11)***	1.72	1.94	2.19
*Number of symptoms*				
Many vs. Few	.99 (.07)***	2.54	2.68	2.82
*Age*				
Adolescent vs. Child	.16 (.07)*	1.03	1.17	1.31
*Problem type x Number of symptoms*				
ADHD (Many vs. Few)	.85 (.18)***	1.99	2.34	2.69
GAD (Many vs. Few)	−.49 (.15)***	.32	.61	.90
ASD (Many vs. Few)	3.97 (.24)***	52.52	52.99	53.46
CD (Many vs. Few)	.49 (.17)**	1.30	1.63	1.96
MDD (Many vs. Few)	1.40 (.18)***	3.71	4.06	4.41
GAD (Many vs. Few)^a^	−1.31 (.23)***	−.18	.27	.72
GAD (Many vs. Few)^b^	−4.34 (.27)***	−.52	0.01	.54
GAD (Many vs. Few)^c^	−.93 (.22)**	−.04	.39	.82
GAD (Many vs. Few)^d^	−1.84 (.23)***	.22	.16	.54
ASD (Many vs. Few)^a^	3.03 (.28)***	20.15	20.70	21.25
ASD (Many vs. Few)^c^	3.41 (.27)***	29.43	29.96	30.49
ASD (Many vs. Few)^d^	2.50 (.27)***	11.53	12.06	12.59
MDD (Many vs. Few)^a^	−.53 (.24)*	.12	.59	1.06
MDD (Many vs. Few)^d^	−.91 (.23)*	−.10	.40	.85
*Problem type x Age*				
ADHD (Adolescent vs. Child)	.51 (.23)***	1.22	1.67	2.12
GAD (Adolescent vs. Child)	−.39 (.18)***	.33	.68	1.03
ASD (Adolescent vs. Child)	−1.23 (.23)***	−.16	.29	.74
MDD (Adolescent vs. Child)	1.73 (.18)***	5.29	5.64	5.99
ASD (Adolescent vs. Child)^e^	1.53 (.28)***	4.07	4.62	5.17
ASD (Adolescent vs. Child)^f^	1.01 (.27)***	2.19	2.72	3.25
ASD (Adolescent vs. Child)^g^	1.40 (.26)***	3.00	4.01	4.52
ASD (Adolescent vs. Child)^h^	2.91 (.27)***	17.64	18.17	18.70
MDD (Adolescent vs. Child)^e^	−1.37 (.25)***	−.24	.25	.74
MDD (Adolescent vs. Child)^f^	−1.90 (.24)***	−.32	.15	.62
MDD (Adolescent vs. Child)^g^	−1.50 (.23)***	−.23	.22	.67
*Problem type x Number of symptoms x Age*				
ASD (Many vs. Few) child^i^	1.96 (.44)***	6.23	7.10	7.96
ASD (Many vs. Few) child^j^	1.96 (.44)***	6.23	7.10	7.96
ASD (Many vs. Few) child^k^	2.03 (.44)***	6.75	7.61	8.47
ASD (Many vs. Few) child^l^	1.96 (.44)***	6.23	7.10	7.96
MDD (Many vs. Few) child^i^	−.74 (.37)*	−.25	.48	1.21
MDD (Many vs. Few) child^j^	−.74 (.37)*	−.25	.48	1.21
MDD (Many vs. Few) child^k^	−.74 (.37)*	−.25	.48	1.21

* *p* < .05, ** *p* < .01, *** *p* < .001

Reference category: ^a^ADHD (Many vs. Few), ^b^autism (Many vs. Few), ^c^CD (Many vs. Few), ^d^MDD (Many vs. Few), ^e^ADHD (Adolescent vs. Child), ^f^GAD (Adolescent vs. Child), ^g^CD (Adolescent vs. Child), ^h^MDD (Adolescent vs. Child), ^i^ADHD (Many vs. Few) Adolescent, ^j^GAD (Many vs. Few) Adolescent, ^k^CD (Many vs. Few) Adolescent, ^l^MDD (Many vs. Few) Adolescent

The number of symptoms presented in the vignettes influenced the frequency of HCPs’ recognition. Vignettes describing problems with many symptoms were recognised more often than those describing few symptoms, *χ*^*2*^ (1) = 194.64, *p* < .001. HCPs recognised 71 % of vignettes with many symptoms whilst 52 % of vignettes with few symptoms were recognised.

**Fig. 1:**
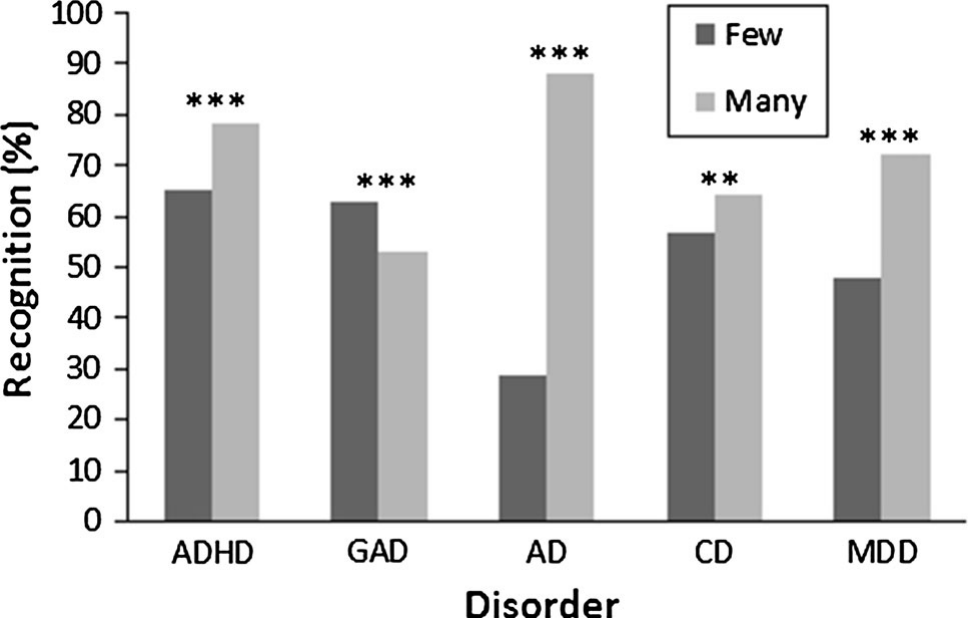
Two-way interaction (problem type x number of symptoms) effects on HCP recognition of mental-health problems. **p < .01, ***p < .001

The effect of problem type on the frequency of HCPs’ recognition differed between vignettes with few and many symptoms, *χ*^*2*^ (4) = 273.29, *p* < .001, see Fig. 1. Firstly, vignettes were more likely to be recognised when describing many symptoms of ADHD, *χ*^*2*^ (1) = 23.03, *p* < .001, ASD, *χ*^*2*^ (1) = 262.12, *p* < .001, CD, *χ*^*2*^ (1) = 8.85, *p* < .01 and MDD, *χ*^*2*^ (1) = 63.21, *p* < .001, than when describing few symptoms of those disorders. Surprisingly, vignettes describing many symptoms of GAD were *less* likely to be recognised than those describing few symptoms of GAD, *χ*^*2*^ (1) = 11.05, *p* < .001. Secondly, vignettes describing many symptoms of GAD were also less likely to be recognised than those describing many symptoms of ADHD, *χ*^*2*^ (1) = 31.75, *p* < .001, ASD, *χ*^*2*^ (1) = 259.97, *p* < .001, CD, *χ*^*2*^ (1) = 17.77, *p* < .01, and MDD, *χ*^*2*^ (1) = 63.81, *p* < .001. In addition, vignettes with many symptoms of ASD elicited *most* recognition and those with few symptoms of ASD elicited *least* recognition in comparison to ADHD, *χ*^*2*^ (1) = 120.64, *p* < .001, GAD, *χ*^*2*^ (1) = 260.55, *p* < .001, CD, *χ*^*2*^ (1) = 162.94, *p* < .001, and MDD, *χ*^*2*^ (1) = 82.93, *p* < .001, vignettes with many and few symptoms respectively. Finally, MDD vignettes with few symptoms were less recognised than few symptom CD vignettes, whilst those with many symptoms of MDD were better recognised than CD vignettes with many symptoms, *χ*^*2*^ (1) = 15.76, *p* < .001. Both few and many symptom MDD vignettes were less recognised than their ADHD equivalents, *χ*^*2*^ (1) = 4.87, *p* < .05. See Table 2 for descriptive statistics and Table 3 for inferential statistics.

### Effect of Demographic Characteristics on Recognition

The age of the child presented in the vignettes influenced the frequency of HCPs’ recognition. Vignettes describing adolescents were slightly, but significantly more likely to result in recognition than those describing children, *χ*^*2*^ (1) = 5.18, *p* < .05. HCPs recognised 60 % of vignettes describing problems in children and 63 % of vignettes describing problems in adolescents, see Table 3 for inferential statistics. There was no effect of ethnic background, *χ*^*2*^ (4) = 0.86, *ns*, gender, *χ*^*2*^ (1) = .49, *ns*, or SES, *χ*^*2*^ (1) = 1.08, *ns,* on HCP recognition.

**Fig. 2:**
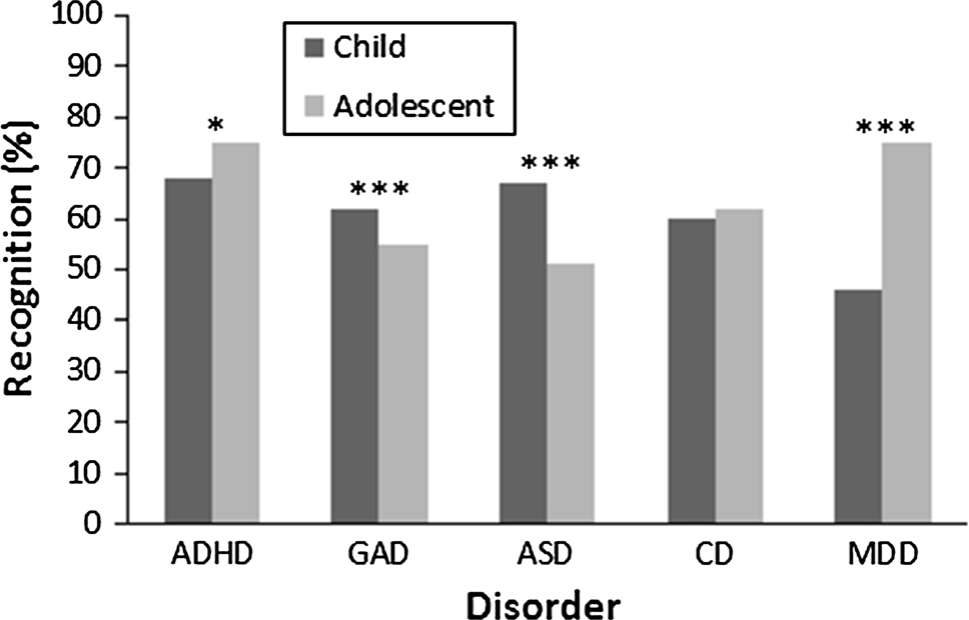
Two-way interaction (problem type x age) effects on HCP recognition of mental-health problems

The effect of problem type on the frequency of HCPs’ recognition differed between vignettes presenting children and adolescents, *χ*^*2*^ (4) = 127.89, *p* < .001, see Fig. 2. Firstly, vignettes were less likely to be recognised when adolescents were described with GAD, *χ*^*2*^ (1) = 5.00, *p* < .001 and ASD, *χ*^*2*^ (1) = 29.28, *p* < .001, than when children were described with those disorders. This difference was greater for ASD than GAD vignettes, *χ*^*2*^ (1) = 13.94, *p* < .001. The variation in recognition of adolescents and children with ASD differed from that of ADHD, *χ*^*2*^ (1) = 30.16, *p* < .001, CD, *χ*^*2*^ (1) = 29.06, *p* < .001 and MDD, *χ*^*2*^ (1) = 116.58, *p* < .001. Specifically, adolescent vignettes were *more* likely to be recognised when described with ADHD, *χ*^*2*^ (1) = 4.95, *p* < .05 and MDD, *χ*^*2*^ (1) = 96.20, *p* < .001, than child vignettes where those disorders were described. Recognition of adolescent and child vignettes describing CD did not vary, *χ*^*2*^ (1) = .88, *p* = *ns*. Finally, the variation in recognition of adolescents and children with MDD differed from that of ADHD, *χ*^*2*^ (1) = 29.39, *p* < .001, GAD, *χ*^*2*^ (1) = 61.53, *p* < .001 and CD, *χ*^*2*^ (1) = 42.46, *p* < .001. Vignettes describing children with MDD were less likely to be recognised than vignettes describing children with all other disorders. See Table 2 for descriptive statistics and Table 3 for inferential statistics.

**Fig. 3:**
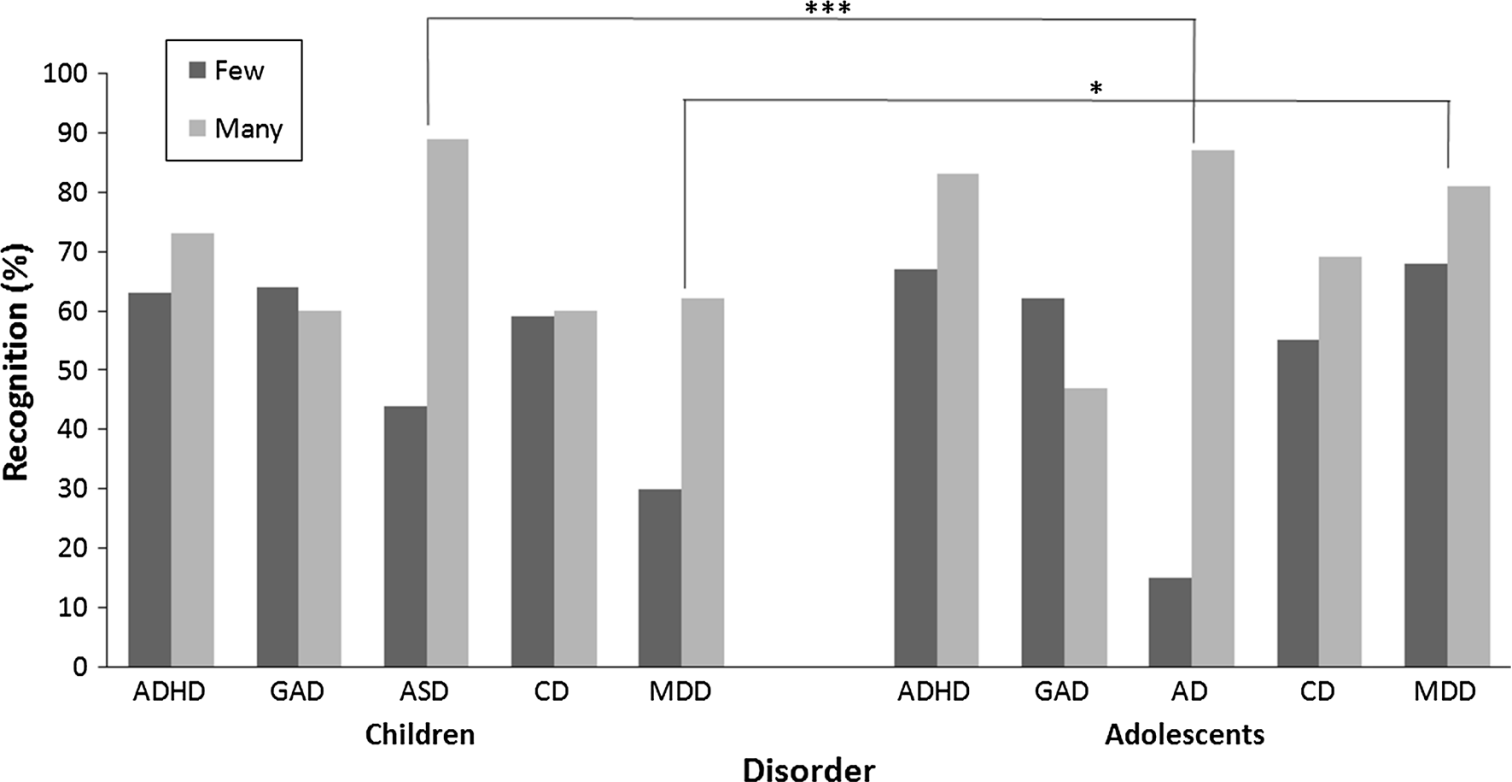
Three-way interaction (problem type x number of symptoms x age) effects on HCP recognition. *p < .05, ***p < .001

Adding a three-way interaction term revealed that the reported *Problem type x Number of symptoms* interaction effects on HCP recognition differ according to the *age* of the child described in the vignette, *χ*^*2*^ (4) = 30.05, *p* < .001, see Fig. 3. The variation in recognition of child and adolescent vignettes describing few and many symptoms of ASD differed from that of ADHD, *χ*^*2*^ (1) = 19.51, *p* < .001, GAD, *χ*^*2*^ (1) = 19.52, *p* < .001, CD, *χ*^*2*^ (1) = 21.23, *p* < .001 and *χ*^*2*^ (1) = MDD, 19.44, *p* < .001. Child vignettes describing many symptoms of ASD were more likely-, whilst adolescent ASD vignettes with few symptoms were less likely than any other disorder to be recognised. Moreover, the variation in recognition of child and adolescent vignettes describing few and many symptoms of MDD also differed from that of ADHD, *χ*^*2*^ (1) = 4.09, *p* < .05, GAD, *χ*^*2*^ (1) = 4.07, *p* < .05, and CD, *χ*^*2*^ (1) = 4.10, *p* < .05. Child vignettes describing few symptoms of MDD were less likely than any other disorder to be recognised. See Table 2 for descriptive statistics and Table 3 for inferential statistics. *Problem type x Number of symptoms* interaction effects on recognition did not differ by *ethnic background*, *χ*^*2*^ (16) = 19.10, *p* = .*ns*, *gender*, *χ*^*2*^ (4) = 2.65, *p* = .*ns* or *SES* of the child presented in the vignette, *χ*^*2*^ (4) = 2.97, *p* = .*ns*.

## Discussion

The aims of this study were to compare HCPs’ recognition of the most common child and adolescent problems, to examine how the number of presenting symptoms influences that recognition and to explore the effect of basic demographic characteristics on recognition of those problems. Although there is clearly room for improvement, HCPs’ general recognition was satisfactory, with approximately sixty percent of all problems recognised. As expected, ADHD was better recognised than any other disorder, whilst GAD was least recognised, and problems which presented many symptoms were recognised more than those with few symptoms. The impact of number of symptoms presented differed between problem types. Of the basic demographic characteristics investigated, only *age* altered HCPs ability to recognise mental-health problems. Adolescents with problems were slightly more likely to be recognised than children. Various interaction effects for the relationship between problem/disorder type, number of symptoms and age will be discussed below.

It is not possible to infer that either the prevalence of ADHD, or its externalising features can be credited for its superior recognition, since CD, which has similar prevalence and externalising features, was no better recognised than GAD, ASD, or MDD. Two-way interaction effects between problem-type and number of symptoms revealed that amongst vignettes describing *few* symptoms, ADHD was also best recognised, and CD better recognised than MDD. Whilst these results show some support for better recognition of the most prevalent and externalising problems, ASD presents externalising features, yet recognisability of vignettes with few symptoms of ASD was extremely poor. This could be related to its low prevalence (CDC, n.d.), which may minimise HCP’s clinical exposure to this problem. With that said, the latter result contrasted enormously with the remarkable recognition of ASD vignettes with many symptoms whereby ASD was *best* recognised despite its low prevalence. Perhaps recent media attention in combination with externalising features can best account for the superior recognition of ADHD across vignettes and ASD in vignettes describing many symptoms. ADHD and ASD have reportedly increased in prevalence in recent years (CDC 2011, 2012), which has, in turn, received media attention (Matson and Kozlowski 2011). This may have added to greater awareness of symptoms amongst the general public, heightened help-seeking, and better awareness of diagnosticians (Matson and Kozlowski 2011).

Superior recognition of ADHD and ASD may, additionally, be due to the distinctive domains featured in these disorders. Symptoms of ADHD and ASD are structured into two and three domains respectively, as opposed to other disorders where all symptoms belong to a single domain (APA 2000). Disorders may be more distinctly problematic, and therefore easiest for HCPs to recognise, when symptoms obviously reflect multiple domains of impairment. Moreover, symptoms of ASD are qualitatively different from behaviours shown by normally developing children, as opposed to symptoms that are found on the ‘normal’ spectrum but to a lesser extent, such as anxiousness in anxiety, or hopelessness in depression (APA 2000). As hypothesised, it seems that just a few core characteristics from each distinct domain might be sufficient to provoke recognition of ADHD, whereby vignettes were best recognised amongst those with few symptoms. However, this idea does not hold for recognition of ASD. Though distinct domains may aid recognition of ASD, it is clear that many symptoms are required in order for HCPs to recognise this problem.

The difference in recognition between few and many symptom (particularly ASD) vignettes demonstrates how increased numbers of presented symptoms *can* increase problem salience and eliminate other potential disorders (McConaughy 2013). However, the number of symptoms presented had differential effects between problems. For ADHD, ASD, CD and MDD, vignettes describing many symptoms led to better recognition than those with few symptoms. In the case of GAD, vignettes describing few symptoms were *better* recognised than those with many symptoms. This result was particularly meaningful since increased information could be expected to aid in recognition of internalising disorders to a greater extent than externalising disorders given their inhibited nature (Mesman and Koot 2000) and symptoms which can occur as part of a ‘normal’ response to external stressors (Beesdo et al. 2009). Indeed recognition of MDD vignettes was certainly improved by increased symptoms. It is possible that reluctance as opposed to recognition is accountable for poor recognition of GAD vignettes with many symptoms. For instance, the DSM-5 advises HCPs that GAD may be over-diagnosed in children and thus to exercise caution when considering its diagnosis (APA 2013). However, GAD is reported to be poorly recognised in general, perhaps because of its similarities with other disorders (Allgulander 2006; Wittchen et al. 2002); and given that children often report physical symptoms when experiencing anxiety, it is often mistaken for a medical problem (Allgulander 2006). Subsequent studies will examine HCPs’ responses to GAD vignettes that were *not* recognised to shed light on the type of problem GAD is considered as being similar to. In fact, responses to *all* unrecognised vignettes could reveal errors in recognition per disorder, as well as any patterns in error.

To our knowledge, this study is the first to examine age effects on HCPs’ recognition of specific childhood mental-health problems. Interestingly, recognition across mental-health problems was slightly but significantly better in adolescents than children. Furthermore, a two-way interaction between problem-type and age revealed that the effect of age differed per disorder. Noteworthy findings include, but are not limited to, better recognition of ADHD and MDD in adolescents than children with those disorders. However, children with ADHD were also better recognised than children with any other disorder, whilst children with MDD were least recognised than children with any other disorder. Conversely, ASD was better recognised in children than in adolescents.

It is not immediately obvious why certain disorders are better recognised in children or adolescents. A review of relevant literature revealed just one study showing that children were also less likely to be recognised with symptoms of any form of psychopathology than adolescents (Kelleher et al. 1997). However, the prevalence of mental-health problems differs for children and adolescents and also varies by problem type. The prevalence of depression in children under 13 is 2.8 and 5.6 % in 13–18 year olds (Costello et al. 2006). It is also reported to be the greatest problem amongst American adolescents (World Health Organization (WHO) 2014). Moreover, whilst the DSM-5 states that MDD can appear at any age, it advises that the likelihood of onset increases with puberty, and only provides information about the disorder occurring from adolescence onwards (APA 2013). Adolescents may therefore be considered a more ‘typical’ case for this disorder which, in turn, may reduce HCPs’ likelihood of considering it an option in children. Similarly, ASD is a disorder which typically begins in infancy and is diagnosed between ages 3 and 7 years (CDC 2012). This may have resulted in HCPs considering an undiagnosed case of ASD in adolescence as unlikely. Three-way interaction effects between problem-type, number of symptoms and age reveal that the poor recognisability of MDD in children and ASD in adolescents is hampered further when few symptoms of these disorders are present. Implications for clinical practice are discussed below.

Surprisingly, HCPs’ recognition of mental-health problems was unaffected by ethnicity, gender and SES. This result defied expectations, as well as some previous findings (e.g., Burke et al. 2015; Cuccaro and Wright 1996; Froehlich et al. 2007) that have shown each of these demographic characteristics to influence recognition. However, previous studies may not have examined the effect of each demographic characteristic whilst controlling for potential effects of the others. For instance, in one study children from an ethnic-minority background were found less likely to have symptoms of autism recognised than their majority-group counterparts (Burke et al. 2015); however, that study did not measure or control for the potential effect of SES. The design used in the present study, exercised tight control over all independent variables; this allows us to conclude that it is likely problem type, number of symptoms and age of a child, above and beyond other demographic characteristics, that influence recognition of mental-health problems. This conclusion is in line with Pescosolido and colleagues (2008), who reported that the *behaviours* rather than demographic characteristics described in their vignettes appeared to drive respondents’ recognition.

The results discussed have implications for clinical practice as well as for children and families of children with mental-health problems. To begin with, it is now apparent that some disorders are easier recognised than others; some disorders need but a few symptoms for recognition whilst others need many. It is therefore important for HCPs to be alert for *all* symptoms of childhood problems that may pose a risk of further developing into problems causing greater impairment (Nelson et al. 2003). All HCPs should be advised in particular about the risk of overlooking ASD and MDD when few symptoms are presented and GAD when many symptoms are presented. Likewise, they should also be informed about superior recognition of ADHD, and ASD when many symptoms are present. In doing so, care must be taken to avoid this knowledge disproportionately influencing HCPs’ subsequent recognition. Similarly, although HCPs should be made aware that children can present with symptoms of MDD and adolescents can have undiagnosed ASD, premature diagnoses of MDD in children are undesired. Finally, delays in HCP recognition may lead to longer diagnostic processes than necessary, longer waiting-lists and unnecessary costs which are frustrating for HCPs and parents and children with mental-health problems alike.

### Limitations

Our sample of HCPs consisted of 90 % women and whilst mental-health care is generally a female dominant profession (Marsella 2011), it would be interesting to see if the reported results extend to men working in the field. The sample also covered a diverse group of relevant HCPs, some of whom may be better than others at recognising childhood mental-health problems. It was however, not within the scope of the current study to examine potential differences between the various types of HCPs, but those results are being reported elsewhere. HCP type was controlled for in all analyses.

In addition, although HCPs often make evaluations about children based on information from the patients’ file (McConaughy 2013), the degree to which results from this study directly transfer to HCP recognition in a clinical setting is unknown. A strength of an analogue design is that it prevents HCPs from being visually and aurally disturbed by symptoms presented. This same strength could have hampered the physical experience of patient symptoms and thus prevented HCPs from fully gauging the problematic nature of the behaviours. Furthermore, whilst symptoms in vignettes were systematically selected, the inclusion of 5 and 10 symptoms resulted in differences in the extent to which criteria were met within each disorder. That is, vignettes with 5 symptoms were at the threshold for diagnosis in some disorders (CD and GAD), but subclinical in other disorders (ADHD, ASD and MDD). Similarly, when 10 symptoms were described, GAD and CD vignettes provided information in excess of what is necessary for a diagnosis whereas ADHD, ASD and MDD vignettes only just qualified for a diagnosis. However, this limitation is unlikely to have affected the results since, for example, GAD with many symptoms had the worst recognition despite the excess information. Finally, the vignettes included in this study did not address comorbidity whilst comorbidity of child and adolescent mental-health problems is extremely common. One implication of this limitation is that, in practice, HCPs’ recognition may actually be worse than has been reported in the current study, given that comorbid problems tend to complicate identification of any individual problem.

## Appendix: Examples of vignettes presented to HCPs

**Example 1**

**Problem type:**: ADHD
**Number of symptoms:**: Many
**Ethnicity:**: Dutch
**Gender:**: Male
**Age:**: Child
**SES:**: Low

Bram is in the third year of primary school. He is seven and a half years old and lives with his parents Bert and Elske and his brother Job in Utrecht, in the house that his dad grew up in. Bram’s parents both work as a postman/woman. They’re worried about him because he becomes agitated about the smallest things. He also gets very easily frustrated when he needs to do something that requires a lot of attention and his hectic behaviour is unmanageable. At home Bram seems to enjoy disrupting his brother’s activities. In addition to this he talks endlessly and frequently interrupts others’ conversations. He doesn’t seem to be able to control himself, even if he tries. This description of him corresponds with the way he is described by his teacher. She has reported Bram to be very easily distracted in comparison to his peers. Providing him with plenty of structure and organisation helps him to finish his work more often.

**Example 2**

**Problem type:**: GAD
**Number of symptoms:**: Few
**Ethnicity:**: Indian
**Gender:**: Female
**Age:**: Adolescent
**SES:**: Low

Jaishree has just turned 15 years old and is in year 10 at secondary school. She lives with her mum, dad and little sister in Venlo. Her dad works as a security guard and her mum is a housewife. Jaishree’s parents are of Indian descent but met each other in Amsterdam where they were both born and bred. Multiple teachers have indicated that Jaishree is cooperative and hardworking but that her constant worries about everyday things are hindering her functioning at school. Jaishree worries excessively about her health and her future. She needs constant reassurance that she’s doing well. Jaishree’s parents also have difficulties reassuring her and don’t know how to deal with her worries.

**Example 3**

**Problem type:**: Autism
**Number of symptoms:**: Few
**Ethnicity:**: Moroccan
**Gender:**: Male
**Age:**: Child
**SES:**: High

Ahmed has just turned 3 years old and is the oldest of three children in a nuclear family of Moroccan descent. The family originally lived in Krimpen aan den Ijssel where Ahmed’s parents were both raised, but moved to Tilburg a year ago for work. Ahmed’s dad is a GP and his mum is a housewife. Ahmed currently goes to preschool for 3 days a week. The teacher there is worried about his language development because it is delayed in comparison to the other children of his age. He hardly speaks unless he is spoken to and even then he has clear difficulty pronouncing simple words, which makes him difficult to understand. Ahmed often sits alone in the playroom absent-mindedly imitating the sounds of the other children.

**Example 4**

**Problem type:**: CD
**Number of symptoms:**: Many
**Ethnicity:**: Turkish
**Gender:**: Male
**Age:**: Adolescent
**SES:**: Low

Serkan is 15 years old and an only child. He lives with his parents Ahmet and Beyza in Rotterdam. His parents immigrated as children to Holland from Turkey. They work as toilet assistant and houseman. Serkan is in year eleven and currently attends the school where his parents met each other when they were 5 years old. Serkan’s parents were recently invited by his head teacher to come and discuss Serkan’s behaviour. Serkan’s parents had been expecting the invitation for a while. During the meeting it emerged that Serkan behaves very aggressively at school. He threatens others in the class and is often intimidating in order to get what he wants. This behaviour sometimes leads to physical violence whereby Serkan cannot be calmed. The head teacher has received multiple complaints from other parents regarding this problem. In addition to this, Serkan often destroys things. In the past year he has caused a lot of damage at school. For example, he intentionally tried to start a fire behind the school building. Finally, the teacher suspects that some missing objects were possibly stolen by Serkan.

**Example 5**

**Problem type:**: MDD
**Number of symptoms:**: Few
**Ethnicity:**: English
**Gender:**: Female
**Age:**: Child
**SES:**: High

Kate is 6 years and 5 months old and in the second year of primary school. She currently lives with her mum Kathleen and dad Mark in Groningen. Kate’s parents originally come from England but have lived their whole lives in Holland. They work as a pharmacist and houseman. Kate has always been sensitive but she was a happy and energetic toddler. Kate’s parents have recently started to worry because she seems to be continuously irritated and can’t shake her negative feelings off. Her teacher has says that Kate has lost all interest and never finishes her work anymore.
